# Differential Expression Pattern of the Human Endoderm-Specific Transcription Factor Sox17 in Various Tissues and Cells

**Published:** 2012-11-01

**Authors:** Ayatollahi M., Sanati M. H., Kabir Salmani M., Geramizaeh B.

**Affiliations:** 1 *Transplant Research Center, Shiraz University of Medical Sciences, Shiraz, Iran*; 2 *National Institute of Genetic Engineering and Biotechnology, Tehran, IranAbstract*

**Keywords:** Sox17, Liver transcription factor, Endoderm formation, Human

## Abstract

Background: Sox17 is a member of the Sry-related high mobility group (HMG) of transcription factors that is necessary for endodermal formation and liver development in multiple species. Sox17 gene expression is required for formation of definitive endoderm that gives rise to various tissues.

Objective: To examine the expression of Sox17 in various human tissues and cells.

Methods: Semiquantitative polymerase chain reaction (RT-PCR) was used to evaluate the expression of Sox17 in adult liver, small intestine, spleen, placenta, fetal liver as well as embryonic stem cells (ESCs), and human HepG2 hepatoma cell line.

Results: Low Sox17 gene expression was observed in ESCs. However, there was no expression of Sox17 in human placental tissue, small intestine, adult liver, spleen, and HepG2 cells. But its expression in human fetal liver was very high.

Conclusion: The data presented in this study reflect the differential expression pattern of Sox17 in the fetal development during early mammalian endodermal formation which is temporal and tightly regulated.

## INTRODUCTION

Sox proteins (Sry-like HMG box gene) were originally defined almost one decade ago with the identification of Sry (sex determining region of Y chromosome) as the gene determining testis development in mice and men [1]. In the course of gene cloning studies in mouse, four other Sox genes expressed during embryogenesis, were identified (Sox1-4). Subsequently, it has led to the identification of 24 different Sox genes in mouse, most of which are as yet uncharacterized [2,3].

Sox genes are known by a conserved DNA sequence encoding an almost 80-amino-acid domain responsible for sequence-specific DNA binding by means of a high-mobility group (HMG) domain, allowing them to function as transcription factors. The HMG domain forms an L-shaped module composed of three helices, and binds to DNA in the minor groove [3,4]. This domain is highly conserved among Sox proteins, and all SOX factors appear to recognize a similar binding motif, A/TA/TGAA/TG. However, protein sequences outside the HMG domain are variable. According to the sequence similarity and genomic organization, the Sox proteins can be divided into 10 groups [2-4].

All the Sox proteins are necessary for many developmental processes including gastrulation and organogenesis [5,6]. Sox2 is required for epiblast and extraembryonic ectoderm formation in the mouse embryo. Sry and Sox9 are required for testis formation and for differentiation of Paneth cells in the intestinal epithelium [7-9]; Sox18 has been shown to be necessary for cardiogenesis and angiogenesis [10].

Sox17 has been found to be necessary for endoderm formation, liver development, and hepatocyte differentiation in several species [11-13]. Sox17 expression is first detected in the extraembryonic endoderm at embryonic day (E)6 and in the anterior primitive streak, which gives rise to the definitive endoderm, by E7 [12,13]. The requirement for Sox17 in endoderm formation was confirmed in another study using morphogenesis. In mice, targeted disruption of Sox17 results in embryonic lethality due to a lack of definitive endoderm formation and liver development [13].

Because definitive endoderm contributes to formation of various organs including liver, pancreas, thyroid, lung, and intestine, we conducted this study to examine the gene expression of Sox17 in various human tissues and cells.

## MATHERALS AND METHODS

Semiquantitative RT-PCR analysis

Total RNA was extracted from placenta, small intestine, spleen, fetal and adult liver tissues using RNXTM RNA extraction kit (CinnaGen) according to the manufacturer’s instructions. In addition, RNA from the embryonic stem cells (ESCs), and human HepG2 hepatoma cell lines were also studied. Extracted RNAs were then quantified by nano-spectrophotometer (Nano-Drop, ND-1000). The cDNA was synthesized from 2 μg of total RNA using the random hexamer primer RTPL12 kit (Vivantis). PCR was subsequently carried out using Accuprime pfx PCRPremix (Invitrogen). The primer designed by Oligo (ver 6) software. The primer sequences and reaction conditions used in this study are listed in Tables 1 and 2, respectively. Relative band intensities were determined using an image analyzer (CS analyzer software). The levels of target mRNA were normalized to the signal obtained for glyceraldehyde-3-phosphate dehydrogenase (GAPDH) mRNA expression. We used the reference gene for primary setting up as well as checking the RNA extraction and cDNA synthesis. Quality of extracted RNA from different samples were determined according to the OD by the rate of 260/280.

**Table 1:** The primer sequences which was designed in this study

	**Primer Sequence**	Tm	**CG content**	**Base Pair**	size
**Sox17**					
**Forward**	5’CAGGCCTGCAGCGCCATGAGCAGCCCG3’	86.9	74.1	27	190-216
**Reverse**	5’CTGGGGCGGATCCGGGACCTGTCACAC3’	85.4	70.4	27	1470-1444
**GAPDH gene**					
**Forward**	5’TGCCGGTGACTAACCCTGCG3’	61	65	20	102-121
**Reverse**	5’CCTGCAAATGAGCCCCAGCCTT3’	62	59.09	22	538-517

## RESULTS

**Figure 1:**
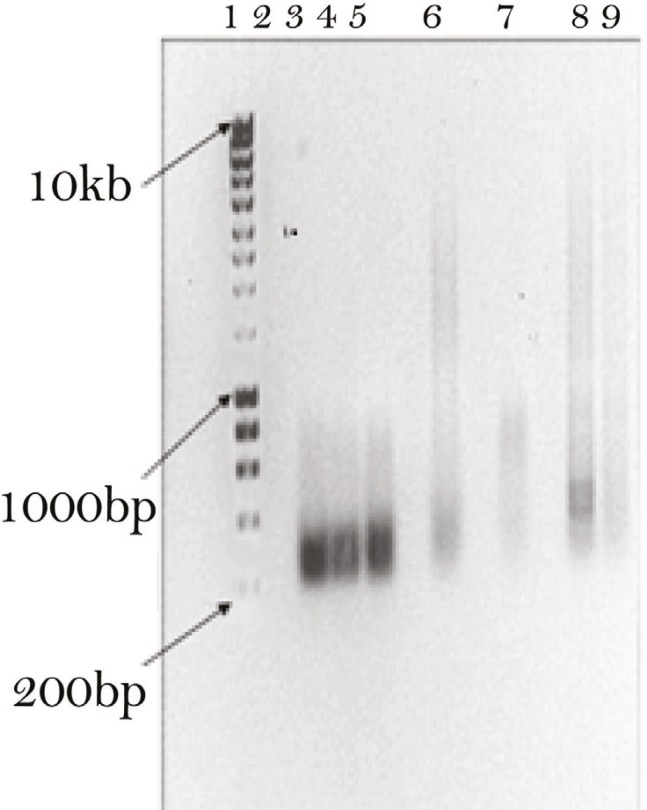
Semiquantitative RT-PCR analysis. Lane 1 is the molecular marker (200–10,000 bp), lanes 3, 4 and 5 show the amplified cDNA from human placenta. Lanes 6, 7, 8, and 9 show the amplified cDNA from human adult liver, small intestine, spleen and HepG2 hepatoma cell line, respectively. Lane 2 shows negative control (without cDNA).

A 1300-kb fragment of the Sox17 mRNA among human tissue and cells was amplified through semiquantitative RT-PCR analysis. Figure 1 shows the PCR products of the amplified cDNA from placental tissue, adult liver, small intestine, spleen and HepG2 hepatoma cell line. As shown in the figure, the agarose gel electerophoresis of all amplified cDNAs were negative.

**Figure 2:**
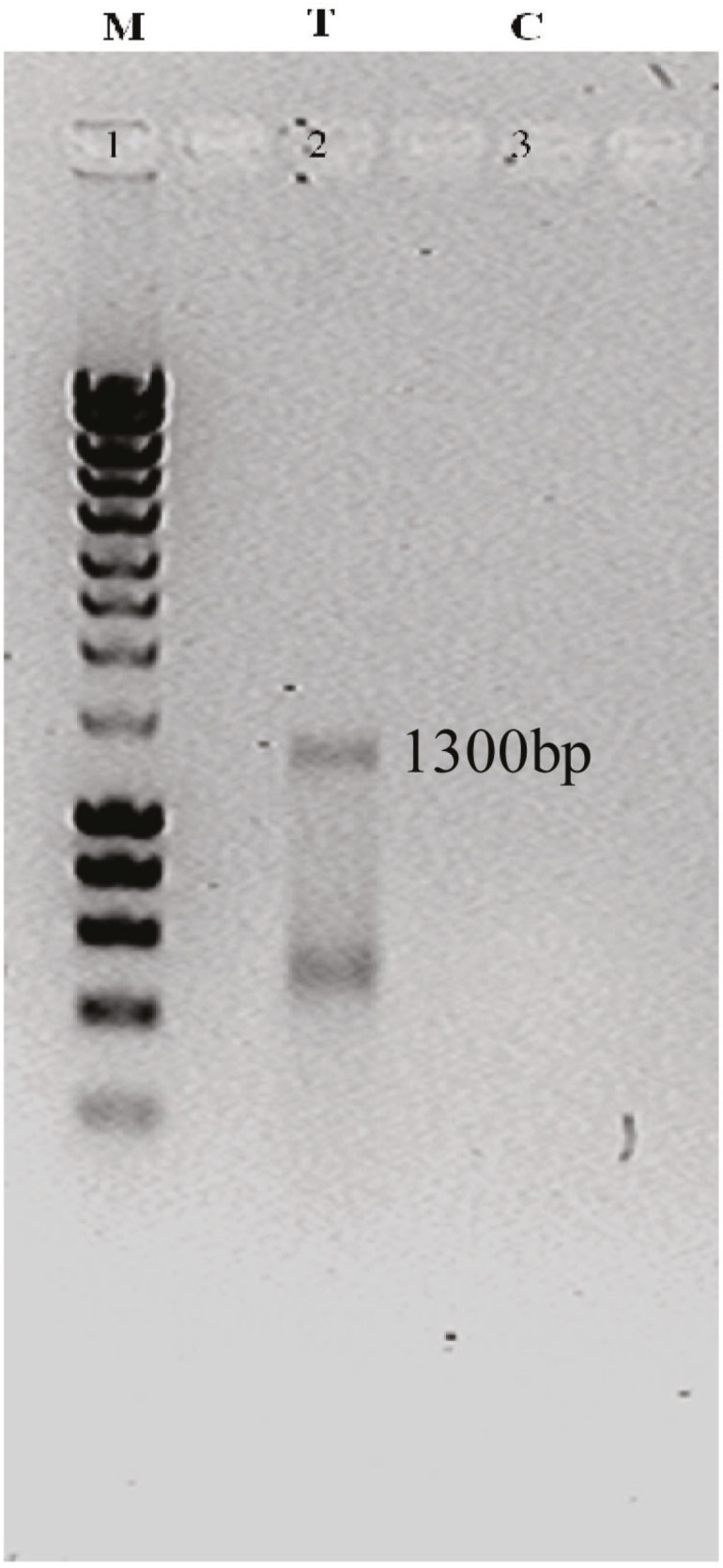
Semiquantitative RT-PCR analysis from fetal liver. Lane M is the molecular marker (200–10,000 bp). Lane T shows a 1300-kb fragment as high expression of Sox17 cDNA in fetal liver. Lane C shows negative control.

**Figure 3:**
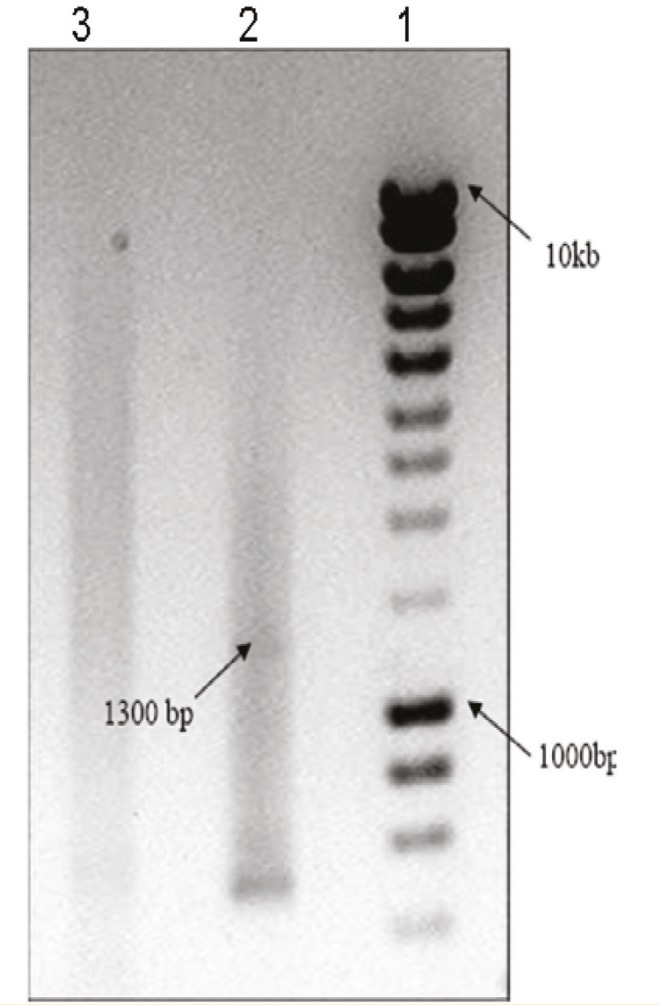
Semiquantitative RT-PCR analysis from embryonic stem cells (ESCs).Lane 1 is the molecular marker (200–10,000 bp). Lane 2 shows a 1300-kb fragment as low expression of Sox17 cDNA in ESCs. Lane 3 shows negative control.

The results from RT-PCR and gel electerophoresis of fetal liver and ESCs showed that the 1300-kb fragment of the Sox17 gene was amplified through RT-PCR analysis. Lane T in Figure 2, shows an abundant expression the Sox17 gene in fetal liver, and lane 2 in Figure 3, shows the low expression of gene in ESCs.

## DISCUSSION

Despite the importance of the Sox family proteins in developmental stages in approximately 30 vertebrate and over a dozen invertebrate Sox genes, the actual role of these proteins and their molecular characterization in various tissues are not well understood and remain an active area under investigation.

Sox17 is a key component of endoderm formation during vertebrate gastrulation. The HMG-box transcription factor, Sox17, binds sequence specifically, to a Sox-consensus motif, and function as a classical liver-transcription factor [13-15].

However, the molecular events controlled by Sox17 are largely unknown. Recent studies have shown that Sox17 and β-catenin cooperate to regulate the transcription of endodermal genes [16].

It has been reported that in the stage of definitive endoderm, cells express Sox17 and GATA-4. Sox17 is required for the formation of liver in several species; GATA-4 is the earliest known transcription factor to bind the albumin gene enhancer in liver precursor cells in embryos [17].

Based on gene expression profiles of fetal *vs*. adult hematopoietic stem cells (HSCs), Jang found that Sox17 was specifically expressed in fetal HSCs, and it becomes undetectable eight weeks after birth [18]. However, Park reported that Sox17 expressed at high levels in the embryonic endoderm; it has also been expressed in mature tissues [19].

As Sox17 is required for formation of definitive endoderm that gives rise to various organs including liver, pancreas, thyroid, lung, and intestine, we examined the expression of the Sox17 in various human tissues including human adult liver, small intestine, and spleen as well as fetal liver, placental tissue, and embryonic stem cells (ESCs). We found that there was no expression of Sox17 in human tissues including small intestine, spleen, placental tissue, and adult liver (Fig. 1). However, low Sox17 gene expression was observed in ESCs (Fig. 3).

We also examined Sox17 gene expression in human HepG2 hepatoma cell line, which was in keeping with the reports that showed Sox17 gene expression in tumor cells including testicular germ cell tumors [20] and all yolk sac tumors [21]. Nevertheless, the gene expression pattern of Sox17 was negative in our study (Fig. 1). We further extend these studies by describing an abundant Sox17 gene expression in human fetal liver (Fig. 2).

Our data suggest that the gene-expression pattern of Sox17 reflects the gene-regulation pattern of transcription factors in the fetal development during early mammalian endodermal formation, which is temporal and tightly regulated. However, a large number of molecular studies still need to be performed to understand the gene expression pattern of Sox17 during early development.
